# Radon Adsorption in Charcoal

**DOI:** 10.3390/ijerph18094454

**Published:** 2021-04-22

**Authors:** Andreas Maier, Jesse Jones, Sonja Sternkopf, Erik Friedrich, Claudia Fournier, Gerhard Kraft

**Affiliations:** 1 Biophysics Department, GSI Helmholtzzentrum für Schwerionenforschung GmbH, 64291 Darmstadt, Germany; Jesse-Jones@live.de (J.J.); sonja.sternkopf@mpibpc.mpg.de (S.S.); efriedrich@ikp.tu-darmstadt.de (E.F.); c.fournier@gsi.de (C.F.); g.kraft@gsi.de (G.K.); 2 Faculty of Physics, Goethe University Frankfurt am Main, 60438 Frankfurt am Main, Germany; 3 Faculty of Physics, Technical University of Darmstadt, 64289 Darmstadt, Germany

**Keywords:** radon, charcoal, adsorption, desorption

## Abstract

Radon is pervasive in our environment and the second leading cause of lung cancer induction after smoking. Therefore, the measurement of radon activity concentrations in homes is important. The use of charcoal is an easy and cost-efficient method for this purpose, as radon can bind to charcoal via Van der Waals interaction. Admittedly, there are potential influencing factors during exposure that can distort the results and need to be investigated. Consequently, charcoal was exposed in a radon chamber at different parameters. Afterward, the activity of the radon decay products ^214^Pb and ^214^Bi was measured and extrapolated to the initial radon activity in the sample. After an exposure of 1 h, around 94% of the maximum value was attained and used as a limit for the subsequent exposure time. Charcoal was exposed at differing humidity ranging from 5 to 94%, but no influence on radon adsorption could be detected. If the samples were not sealed after exposure, radon desorbed with an effective half-life of around 31 h. There is also a strong dependence of radon uptake on the chemical structure of the recipient material, which is interesting for biological materials or diffusion barriers as this determines accumulation and transport.

## 1. Introduction

The naturally occurring radioactive noble gas radon is pervasive in our environment. In areas of elevated uranium concentration, it emanates from the soil [1]. The most abundant isotope is ^222^Rn, which originates from the decay chain of ^238^U. Radon is responsible for the largest proportion of radiation exposure from natural sources and the second leading cause of lung cancer induction after smoking [2]. Depending on several factors such as the uranium content or the gas permeability of the soil, there are large regional fluctuations of the radon activity concentration [3,4]. Therefore, the measurement of the local activity concentrations in buildings and common rooms is highly relevant, especially in areas where elevated radon levels above a reference level of 300 Bq/m^3^ can be expected [2]. For this purpose, a number of different measurement techniques are used, such as electronic devices, which are generally more expensive than the frequently used CR-39 and electret methods [5]. Aside from these commonly used procedures, charcoal can be used as a passive and cheap measurement technique. Radon can bind to charcoal via Van der Waals interaction [6]; therefore, charcoal can be used to determine the activity concentration in buildings accordingly [7]. Additionally, it can be used to determine the permeability of membranes [8]. In most of these measurements, exposure times are in the region of several days, where meteorological conditions such as humidity, temperature and atmospheric pressure can have an influence on radon adsorption [9], especially with longer exposure times [7]. For this reason, it is important to specify the loading capacity of charcoal for radon and potential influencing factors during exposure. Additionally, specification of radon desorption from charcoal is of great interest, as this knowledge is important to determine the correct radon activity concentration in the sample.

## 2. Materials and Methods

In our experiments, charcoal was exposed in a radon chamber. In this experimental setup, various parameters such as temperature, relative humidity and radon activity concentration can be varied and monitored within certain limits. The chamber itself is made of stainless steel and has a volume of 50 L. Radon is provided by a commercially available ^226^Ra-source (RN-1025, Pylon Electronics, Ottawa, ON, Canada) and the radon activity concentration is measured with an active measurement device (RTM 1688–2, Sarad, Dresden, Germany). During exposure, the setup is sealed and all parameters are permanently monitored. Afterward, the whole system is flushed with normal air. Activity concentrations for the experiments presented here were in the range of 9 to 420 kBq/m^3^. A more detailed description can be found in the work of Maier et al. [10]. The commercially available charcoal was made of coconut husk and formed to a cylindrical shape with a diameter of 4 mm and a length of up to 10 mm. Before experiments, the charcoal was baked out for 2 h at 187 °C to avoid contamination by naturally occurring radon gas.

**Figure 1:**
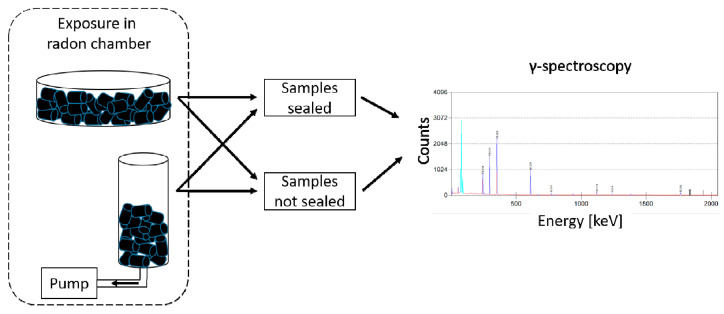
Schematic of the used experimental procedure. Samples were exposed in a radon chamber either in an open Petri dish or in a small cylinder with an attached pump. Afterward, the samples were removed and transferred to other sample containers, which were partly sealed. After at least 4 h, the recording of γ-spectra was started.

Experiments were conducted by placing a certain amount of charcoal either in a Petri dish inside the radon exposure chamber or in a small cylinder (see Figure 1). The latter was attached to a pump with an airflow of 193.4 ± 2.8 mL/min, and the whole system was also placed inside the exposure chamber. During radon exposure, the chamber was sealed; after distinct exposure times, it was flushed with normal air for 5 min. Afterward, the samples were removed and transferred into other sample containers to avoid surface contamination. At this point, some of the containers were sealed, and the others were left open. In both cases, a radioactive equilibrium between radon and its short-living decay products ^218^Po, ^214^Pb, ^214^Bi and ^214^Po could be maintained after around 4 h. In the containers that were not sealed, radon was expected to desorb from charcoal, giving hints on the transport rate. In the sealed containers, the measured activity was expected to decrease with the half-life of radon, which is the standard measurement procedure used.

**Figure 2:**
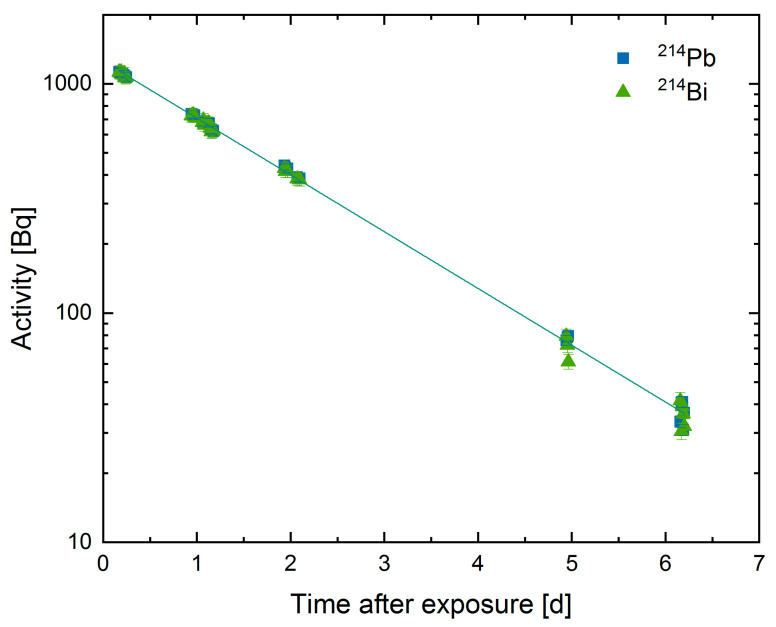
Exemplary dataset for a charcoal sample. Measurements took place up to 6 days after the end of exposure. The solid line is described by a linear fit, which is conterminous with an exponential decay.

Afterward, the decay of the γ-emitting radon progeny ^214^Pb and ^214^Bi was measured with a high purity germanium detector (HPGe). For data analysis, only measurements following a time interval of more than 4 h after the end of exposure were taken into account. The activities were measured over several hours, and the whole experiment took between 5 to 14 days with typical measurement times of 15 min each. Due to the relatively high measured activities, the count rate was sufficient for analysis. The determined γ-activities were plotted over the time after exposure. An exemplary dataset is depicted in Figure 2.

The intersection of the extrapolated activities with the time point zero gives the initial activity of the measured isotope and thus the ^222^Rn activity at the end of exposure. Additionally, the radon activity concentration during the experiments was measured. Details on the analysis procedure can be found in the work of Maier et al. [10] and Sanjon et al. [11]. Finally, the activities could be normalized to the mass of the sample and the radon activity concentration inside the chamber during exposure. In every experiment, activity values for ^214^Pb and ^214^Bi were determined. The presented values are mean values of up to 14 samples, and the error was determined by using Gaussian error propagation.

In total, four different experiments were performed:

- Saturation: A distinct amount of charcoal (6.0 ± 0.1 g) was exposed for 5, 30 and 60 min in the radon chamber. The charcoal was placed in a Petri dish with a diameter of 5.4 cm and a height of around 1 cm (number of samples: *n* = 3).
- Humidity: In order to study the possible influence of the humidity during experiments, charcoal was exposed for 1 h either in a Petri dish or in a small cylinder with an active airflow through the sample at different relative humidity levels ranging from 5 to 94% (*n* = 14).
- Effective half-life: To explore the transport of radon out of charcoal during our measurements, samples were exposed for 1 h and transferred into another open container (*n* = 14). Afterward, measurements, as described above, took place. The radon activity in our sample can be described by the following equation:

(1) R222n(t)dt=−λeff·R222n(t).

Here, the effective decay constant *λ*_eff_ is the sum of the decay constant of radon and the desorption from the sample:

(2) $$\lambda_{\mathrm{eff}}=\lambda_{\mathrm{Rn}}+\lambda_{\mathrm{des}}$$

To calculate the effective half-life, the following equation is used:

(3) T12eff=ln2λeff.

- Bound radon: To determine the amount of radon bound to charcoal, samples were exposed in a small cylinder with an active airflow (*n* = 6). Afterward, the samples were sealed and measured as described before. From the determined initial activities, the number of radon atoms bound to charcoal per unit mass could be calculated. In parallel, the number of radon atoms inside the radon chamber could also be calculated. With this, the percentage of bound radon per unit sample mass could be determined. The same measurement procedure was done for the most abundant fatty acids in the human body, namely oleic acid and linoleic acid, and for isotone solution (0.9% NaCl) for comparison reasons [11].

## 3. Results

### 3.1. Saturation

**Figure 3:**
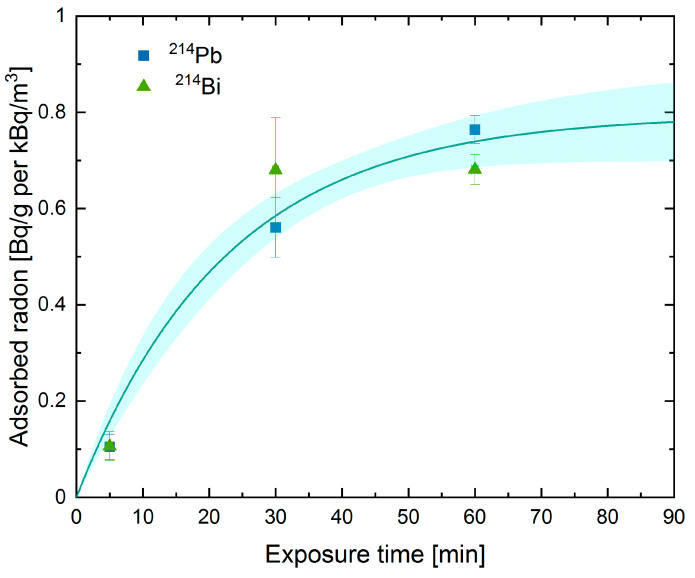
Saturation of charcoal at different exposure times measured for ^214^Pb and ^214^Bi, which are in radioactive equilibrium with ^222^Rn. The solid line represents our fit, described by a restricted growth; the colored area shows the 1σ confidence interval of the fit. For the fitting procedure, the uncertainty was weighted by 1/σ^2^, with σ being the error bar size.

In order to avoid the influence of surface contamination of the Petri dishes, the samples were removed after exposure and transferred into another dish with the same dimensions. Measurements with an HPGe γ-detector were started 4 h after the end of exposure, allowing the establishment of radioactive equilibrium of ^214^Pb and ^214^Bi with ^222^Rn. Additionally, surface contamination of the sample by radon progeny produced during exposure, which could cause a falsely elevated activity, was negligible after this time. From these results, the activity inside the samples at the end of exposure could be determined as a function of the total exposure time and is shown in Figure 3. The fit is described by a restricted growth with a final value of 0.79 ± 0.08 Bq/g per kBq/m^3^. After 60 min, which was the subsequently used exposure time, 94% of the final value was reached.

### 3.2. Humidity

Samples of charcoal were exposed either in a Petri dish or in a small cylinder with an active airflow. During the experiments, a stable humidity inside the radon chamber was maintained, ranging from 5 to 94% between experiments. After exposure, the samples were transferred and measurements with the HPGe detector took place to determine the initial radon activity inside the samples directly after exposure. In both cases, no influence of the humidity was found within the fluctuations of 10.2% for the charcoal activity in the Petri dish and 13.5% for the active exposure.

### 3.3. Effective Half-Life

To explore the transport of radon out of charcoal, samples were not sealed after exposure, allowing radon diffusion from the sample container. With the measured values, the decrease in activity over time can be determined. For our experiments, an effective half-life for radon of T12eff = 1.29 ± 0.42 days could be determined by applying Equations (1)–(3), which is significantly shorter than the physical half-life of 3.82 days. When the samples were sealed, a half-life of 3.79 ± 0.08 days was measured.

### 3.4. Bound Radon

For the passive exposure, we almost reached saturation after 1 h. The same can be expected for the active exposure when a small pump was attached to the sample vessel. Moreover, slightly higher values can be assumed, as in this case, radon was more prone to interact with the surface of the charcoal. Therefore, for these experiments, the amount of adsorbed radon can be calculated as 1.41 ± 0.21 Bq/g per kBq/m^3^.

It is known that radon is likely to be retained in fatty acids, such as the most abundant ones in the human body, linoleic acid and oleic acid, and is retained in much smaller amounts in isotone solution (0.9% NaCl) [11]. To compare the quantity of retained radon in these substances and charcoal, the number of radon atoms per gram sample mass was calculated and divided by the number of radon atoms in the exposure chamber during experiments. The results are given in Table 1. The number of radon atoms per unit charcoal is 2 orders of magnitude higher than in linoleic or oleic acid and 4 orders higher than in isotone solution.

## 4. Discussion

As stated above, there are several applications for radon measurements with charcoal, such as determination of the permeability of membranes [8] or radon activity concentrations in air [9], as it is a cost-effective and easy-to-handle procedure. In most of these measurements, exposure times are in the region of several days, where meteorological conditions such as humidity, temperature and atmospheric pressure can have an influence on radon adsorption [9], especially with longer exposure times [7]. In the data presented here, saturation of radon retention was already reached after around 1 h; however, higher radon activity concentrations than the average naturally occurring concentrations were used. Nevertheless, in some regions, radon activity concentrations of several kBq/m^3^ can be reached [12].

Due to the short necessary exposure time, no influence of humidity could be detected. This is in agreement with the study of Tsapalov et al., in which charcoal was used to measure radon flux from the soil. In their setup, sampling durations of 3–5 h were sufficient and little influence of the weather conditions could be identified [13]. Moreover, Wilson stated that temperature and humidity should be much less important for short sampling periods [6].

When the exposed charcoal was left unsealed after transfer, an effective half-life of around 31 h could be determined, which is similar to the half-life reported in the literature between approximately 20 and 25 h [7]. For a correct evaluation of radon adsorbed to charcoal, this effect is important, as radon will not only decay but also desorb from the sample. Therefore, charcoal devices should not be used for more than 2–4 days, depending on their composition [14]. This fact has a negligible influence on the measurements presented here.

Compared to biologically relevant materials such as fatty acids or isotone solution, radon can be adsorbed more efficiently by charcoal. As charcoal has a large surface area for a given mass, there are many available adsorption sites [6]. The large difference between charcoal and the other materials, as well as the difference between fatty acids and isotone solution, shows a strong dependence of radon uptake on the chemical structure of the recipient material [11,15]. These findings are interesting for biological materials such as different types of tissue, as well as for diffusion barriers.

## 5. Conclusions

In the work presented here, we showed that humidity only plays a negligible role in the adsorption of radon to charcoal for short exposure durations. Additionally, we found that the half-life of radon due to radioactive decay and desorption can be neglected for short exposure durations. As radon was in equilibrium with its progeny in our measurements, the measured activities of ^214^Pb and ^214^Bi allowed direct determination of the ^222^Rn activity. Therefore, the initial activity directly after exposure can be correlated with the radon activity concentration inside the radon chamber and used as a passive measurement device. Moreover, we found that there are large differences in radon adsorption in different materials, depending on their chemical structure; this finding is important for the specification of biological effects or risk associated with radon as these differences determine transport and accumulation in the human body.

## Figures and Tables

**Table 1:** Bound radon atoms in the sample per gram in relation to radon atoms in the radon exposure chamber.

Sample	*N*_sample_(g^−1^)/*N*_chamber_ [%]
Charcoal	(2.83 ± 0.42)
Linoleic acid	(7.98 ± 1.93) × 10^−2^
Oleic acid	(5.60 ± 0.61) × 10^−2^
Isotone solution (0.9% NaCl)	(8.19 ± 1.20) × 10^−4^

## Data Availability

The data presented in this study are available on request from the corresponding author.
